# Dynamic Dysregulation of Ribosomal Protein Genes in Mouse Brain Stress Models

**DOI:** 10.3390/stresses4040061

**Published:** 2024-12-12

**Authors:** Vandana Sharma, Rammohan Shukla

**Affiliations:** 1 Department of Zoology and Physiology, University of Wyoming, Laramie, WY 82071, USA; 2 Department of Neurosciences, University of Wyoming, Laramie, WY 82071, USA

**Keywords:** evolution, ribosomes, stress, synapse, heterogeneity

## Abstract

Emphasizing their evolutionarily conserved role in stress adaptation mechanisms, ribosomal protein genes (RPGs) are observed to be downregulated in various stressors and across phyla. However, this evolutionarily conserved stress response is not well explored in mouse models of neurobiological stress. This study investigates the dysregulation patterns of RPGs in various murine preclinical stress paradigms across different brain regions using available transcriptomic data and identifies the non-canonical ribosomal functions using synaptic gene-ontology terms. Without a discernible structure across different brain areas, we observed heterogeneous dysregulation, encompassing either up or downregulation in both cytoplasmic and mitochondrial RPGs. However, downregulation was more prominent than upregulation, and the overall dysregulation seems more prevalent in the chronic stress paradigm compared to stress paradigms involving acute and early-life stress. Enrichment analysis significantly associates dysregulated RPGs with post-synaptic gene ontology terms, emphasizing their involvement in synaptic modulation. Overall, the study demonstrates ribosomal dysregulation as an evolutionarily conserved stress response mechanism during different mouse stress paradigms. We discuss the possibility that the variability in the directionality of dysregulation may emerge as a potential marker of neuronal activity in response to diverse stress paradigms and the involvement of paradigm-specific RPG dysregulation either in the process of global downscaling of ribosome biogenesis or in the process of ribosomal heterogeneity, each leading to a different effect.

## 1. Introduction

Ribosomes are key players in stress response, primarily orchestrating a homeostatic mechanism involving decreased ribosome biosynthesis to conserve cellular energy and nutrients [1,2]. Showcasing their pivotal role in adaptation, this stress response by ribosomes exhibits significant evolutionary conservation across various species and stressors [3–6]. Ribosomal proteins (RPs) are fundamental to this response. They constitute roughly 6% of the total proteome [7] and are rich in essential amino acids like lysine and arginine [1,8], whose deficiency can result in depression [9–11]. Surprisingly, while extensively studied in lower organisms, scant attention has been given to analyzing ribosomal infrastructure in human depression and in animal models under psychological stress, associated with depression-like states. To address this, in our previous study, we investigated the enrichment of ribosomal protein gene (RPG) families from the Human Genome Nomenclature Committee (HGNC) within the transcriptomic profiles of human prefrontal cortices, including the dorsolateral prefrontal cortex (DLPFC), orbitofrontal cortex (OFC), and anterior cingulate cortex (ACC), as well as the Nucleus Accumbens (NAc), a basal forebrain area, in postmortem human subjects with depression. Additionally, we analyzed the prefrontal cortex (PFC) and NAc of mice exposed to chronic variable stress (CVS), a stress paradigm reflecting various physiological responses to psychological stress [12]. In both paradigms, we observed a significant downregulation of RPGs in the prefrontal regions but not in the NAc.

In murine models, stress can be induced using various protocols beyond CVS, each with distinct biological relevance to disease pathophysiology (Table S1). Variations in stress attributes, such as frequency, duration, intensity, associated pain, and predictability, enable the evaluation of a range of negative behavioral outcomes, including social withdrawal, fear, anxiety-like behavior, and anhedonia. As a result, these protocols are widely used as preclinical models for investigating conditions such as depression and post-traumatic stress disorder (PTSD).

Here, we hypothesized that, being a homeostatic response, RPG downregulation can be observed in other stress paradigms as well. Thus, expanding beyond our initial investigation into CVS, this data-mining study explores the dysregulation of RPGs in available datasets from other murine stress paradigms and their non-canonical enrichment in synaptic pathways using the Synaptic Gene Ontology (SynGO) database—a resource specifically designed to capture gene sets associated with synapse-related functions and processes [13], highlighting the potential involvement of RPGs in synaptic modulation.

## 2. Results

**Figure 1.**
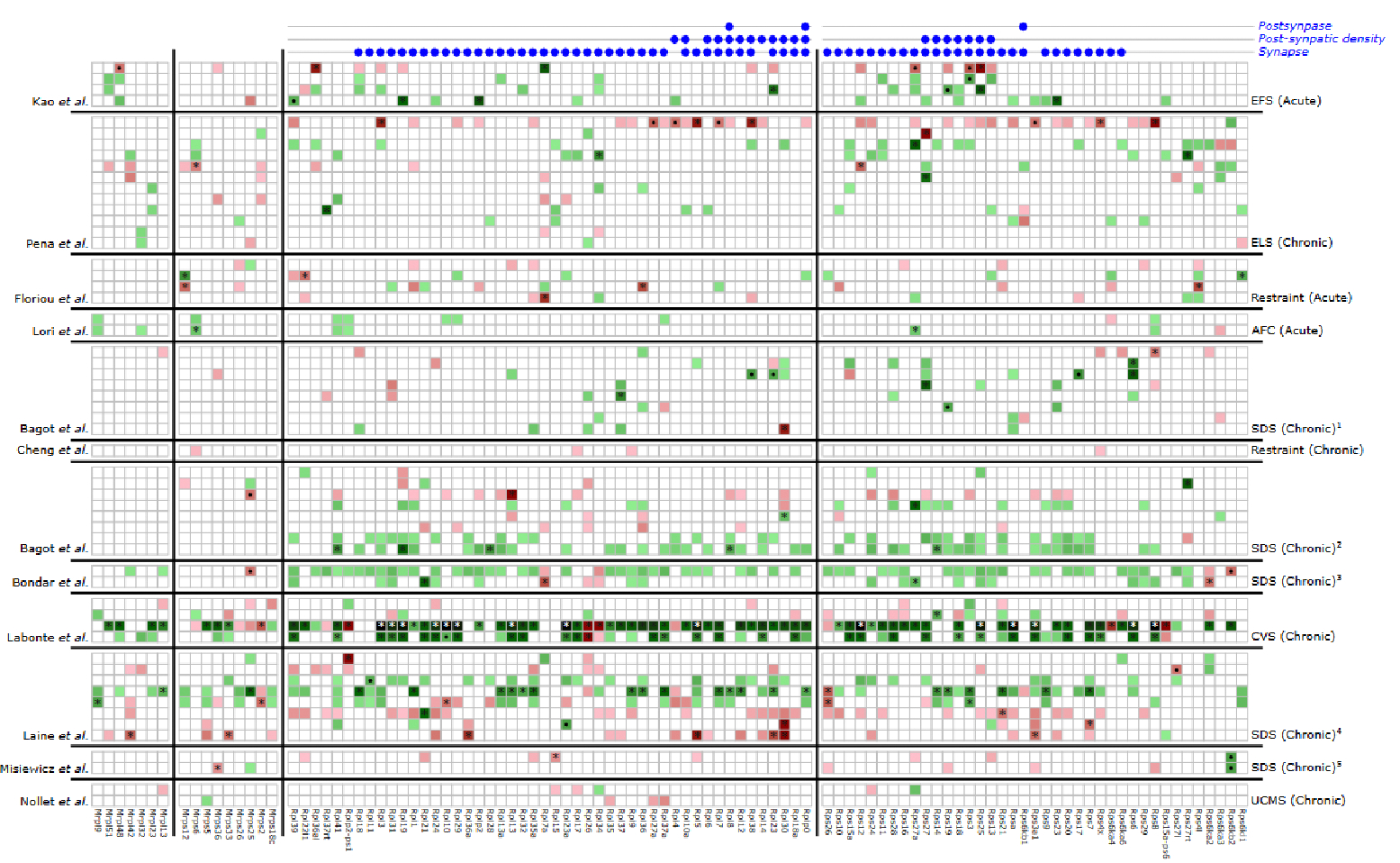
Differential RPG expression across different stress paradigms. The depiction shows the upregulation (in red) and downregulation (in green) of various mitochondrial and cytoplasmic RPGs. White indicates RPGs are not dysregulated in that particular stress paradigm and contrast. The left labels correspond to different studies from which the data was collected [14–25], while the right labels represent various stress paradigms. Enriched synaptic ontology terms are also presented, indicated by labels in the top right. RPGs associated with the enriched synaptic ontology are marked with blue dots at the top. All results with a *p*-value < 0.05 are displayed. Those with an adjusted *p*-value < 0.05 are highlighted with “*”, and those with a *p*-value < 0.001 are highlighted with “•”. Mitochondrial RPGs are identified by names starting with “M.” Stress abbreviations: EFS—electric foot shock; ELS—early life stress; AFC—auditory fear conditioning; SDS—social defeat stress. Time from stress to dissection: ^1^: 28 days; ^2^: 30 days; ^3^: 24 h; ^4^: 6 days; ^5^: 7 days. For details of each stressor and contrast, refer to Tables S1 and S3.

The results are presented in a manually supervised manner, with rows organized by different studies [14–25] and their contrasts and columns representing large and small subunit RPGs of mitochondrial and cytoplasmic origin (Figure 1 and Table S3). Unsupervised hierarchical clustering of rows and columns, along with supervised clustering by brain region (Figure S1 and Table S4) and sex (Figure S2 and Table S5), revealed no distinct patterns, suggesting that the RPG dysregulation is highly heterogeneous and lacks a discernible structure. Intriguingly, with a few exceptions, the number of dysregulated RPGs in chronic stress paradigms was higher than those observed in paradigms involving acute and early-life stress.

There are over 80 cytoplasmic RPGs and a nearly equal number of mitochondrial RPGs in the mouse genome. Our findings reveal that more than half of these genes are dysregulated across various stress paradigms examined in our analysis, with a lower number observed for mitochondrial RPGs compared to cytoplasmic RPGs. Many studies included multiple contrasts, and dysregulation was variable across them. There was variability in the direction of regulation (up or down); however, consistent with the mechanism involving decreased ribosome biosynthesis to conserve cellular energy and nutrients, the downregulation (depicted in green) was more pronounced than the upregulation (depicted in red) (Figure 1).

To explore the role of dysregulated RPGs beyond canonical pathways associated with ribosomal synthesis, protein metabolism, and organelles (specifically mitochondrial RPGs), we conducted an enrichment analysis focusing on synaptic pathways using the SynGO, a database of synaptic gene ontologies. The results indicate that RPGs are significantly associated with postsynapse (q-value = 1.01 × 10^−65^), synapses (q-value = 1.25 × 10^−60^), and postsynaptic density (q-value = 5.85 × 10^−16^), suggesting their involvement in synaptic modulation.

## 3. Discussion

Our results reveal the dysregulation of several RPGs across various stress paradigms. This dysregulation appears heterogeneous, lacking a discernible pattern. However, its enrichment in postsynapse and postsynaptic density implicates broader functional roles beyond the canonical ribosome-associated pathways, shedding light on a significant association with input modulation. Confirming the biological nature of the observed heterogeneity in our results, previous studies investigating the distribution of RPGs in somatic and neuritic compartments of neurons have demonstrated significantly higher enrichment of RPGs in neurites compared to the soma [26–29], accompanied by greater variability in RPG expression within neuritic compartments compared to other gene families [30]. Below, we discuss potential mechanisms through which this variability in RPG expression may contribute to stress and stress-related mood disorders.

Beyond a homeostatic decrease in ribosome biosynthesis during stress, which may trigger a potential global decline in ribosome counts, the homeostatic reduction in RPGs can initiate various other modifications in ribosomal function and structure. For example, ribosome biosynthesis, which relies on equimolar levels of RPs, is sensitive to RP gene dosage [31]; and different stress paradigms leading to differential RPG transcription (Figure 1) could disrupt the molar quantity (stoichiometry) among the core RPs. While ribosomes are typically perceived as a uniform entity, alterations in stoichiometry can lead to the creation of heterogeneous ribosomal populations [32]. These heterogeneous ribosomes may potentially undergo specialization [33], influencing the translation of specific mRNAs or modifying translational efficiency during stress.

We observed variability in the number of dysregulated RPGs in an area- and stress-paradigm-specific manner. However, the number of dysregulated RPGs was higher in the chronic stress paradigm as compared to acute and early life stress. The dysregulation of a larger proportion of RPGs can perhaps lead to a global decrease in ribosome biogenesis, thus decreasing global ribosome numbers. On the other hand, the dysregulation of a few RPGs can lead to heterogeneous ribosomes with an alteration in the stoichiometry of a few ribosomal proteins. Notably, given the enrichment of RPGs in the dendrite (postsynapse) and the known mobility of ribosomes across them, the changes leading to heterogeneous ribosomes can be more local in their effect. Both global and local alterations in RPGs can be a homeostatic process [2]. However, the local changes leading to ribosomal specialization can alter the postsynaptic proteome, leading to site-specific input modulation. In this regard, it is logical to surmise that acute and early life stress can lead to local changes in synaptic input, while chronic changes can lead to a global change in synaptic modulation.

We also observed variability in the directionality of dysregulation, with downregulation being more prominent than upregulation. While additional physiological experiments are needed to validate this mechanism, the homeostatic regulation of RPG expression during stress in synaptic compartments appears to extend to homeostatic synaptic scaling [34]. Homeostatic synaptic scaling functions through negative feedback and aims to restore neuronal activity patterns to their initial set point by adjusting synaptic strengths in the opposite direction [35]. Therefore, the directionality of dysregulation likely depends on the specific neuronal activity patterns associated with different stress paradigms and areas and perhaps could serve as markers of neuronal activity in response to various stress paradigms.

There are a few limitations to this study. Firstly, area- and sex-specific analyses did not reveal any discernible patterns. However, the uneven sample distribution limits the strength of these observations. A more comprehensive and balanced study with sufficient statistical power is needed to draw definitive conclusions regarding the influence of area and sex on RPG expression. Likewise, we reported significance at both *p*-value < 0.05 and adjusted *p*-value < 0.05 to capture broad RPG dysregulation across different stress paradigms. Thus, the results and discussion should be interpreted as hypothesis-generating rather than conclusive.

In summary, ribosomal dysregulation, an evolutionarily conserved stress response mechanism, appears to be active during neurobiological stress, and the observed heterogeneity in dysregulation across different brain areas and stress paradigms reveals an important additional layer of regulation to protein synthesis.

## 4. Material and Methods

### 4.1. Data Processing and Differentially Expressed Genes (DEGs) Selection Method

We utilized the Stress Mouse Portal [(http://hpc-bioinformatics.cineca.it/stress_mice/main) (accessed on 01/06/2024)], a platform that consolidates data from various mouse stressors (Tables S1 and S2) and brain areas [36]. To reduce bias when selecting DEGs across various conditions and datasets, the portal utilizes a standardized pipeline for computing DEGs. This involves comparing samples that differed solely in exposure to stress versus non-exposure while controlling for all other variables like the brain region analyzed, the subject’s strain and sex, or the duration between stress exposure and brain dissection. All contrasts involved comparing stressed animals with non-stressed animals from either different brain regions or sexes. All DEGs with a threshold *p*-value < 0.05 across datasets were filtered for mitochondrial or cytoplasmic ribosomal protein genes and categorized into bins based on significance levels: adjusted *p*-value < 0.05, *p*-value < 0.01, and *p*-value < 0.05 (see Figure 1 and accompanying legend). Only datasets with ≥5 differentially expressed RPGs are included in this study. The detail of each contrast is shown in Table S3.

### 4.2. Calculation of Signed Enrichment Score of Differentially Expressed RPGs

For assessing the strength and directionality of change, we calculated a signed enrichment score using the *p*-value and log-fold change (lfc) values of all differentially expressed RPGs. The formula used is as follows: *ES* = −*log10(p-value) x sign(lfc)*. A heatmap (Figure 1) illustrating the pivot table of enrichment scores, with different datasets as rows and RPGs as columns, was generated using the ComplexHeatmap package in the R programming language. All contrasts studied within datasets were grouped together.

### 4.3. SynGO Enrichment Analysis

To explore the non-canonical functionalities linked with RPGs, we performed an enrichment analysis utilizing the SynGO database [36]. The RPGs that showed significant dysregulation across all studies in the stress portal were compared against the default background list. Three synaptic ontology terms (Figure 1, top) were found to be significantly enriched at a 1% false discovery rate (FDR).

## Supplementary Material

Supplementary Information

Supplementary Tables

## Data Availability

The original data presented in the study are openly available in Stress Mouse Portal (http://hpc-bioinformatics.cineca.it/stress_mice/main).

## References

[1] An H; Ordureau A; Körner M; Paulo JA; Harper JW Systematic Quantitative Analysis of Ribosome Inventory during Nutrient Stress. Nature 2020, 583, 303–309. 10.1038/s41586-020-2446-y. 32612236 PMC7351614

[2] Sharma V; Swaminathan K; Shukla R The Ribosome Hypothesis: Decoding Mood Disorder Complexity. Int. J. Mol. Sci. 2024, 25, 2815. 10.3390/IJMS25052815. 38474062 PMC10931790

[3] Navarro IC; Tuorto F; Jordan D; Legrand C; Price J; Braukmann F; Hendrick AG; Akay A; Kotter A; Helm M; Translational Adaptation to Heat Stress Is Mediated by RNA 5-methylcytosine in Caenorhabditis Elegans. EMBO J. 2021, 40, e105496. 10.15252/EMBJ.2020105496/SUPPL_FILE/EMBJ2020105496-SUP-0002-EVFIGS.PDF. 33283887 PMC7957426

[4] Cheng-Guang H; Gualerzi CO The Ribosome as a Switchboard for Bacterial Stress Response. Front. Microbiol. 2020, 11, 619038. 10.3389/FMICB.2020.619038. 33584583 PMC7873864

[5] Hang R; Wang Z; Deng X; Liu C; Yan B; Yang C; Song X; Mo B; Cao X Ribosomal RNA Biogenesis and Its Response to Chilling Stress in Oryza Sativa. Plant Physiol. 2018, 177, 381–397. 10.1104/PP.17.01714. 29555785 PMC5933117

[6] Sun J; Kim J; Jeong H; Kwon D; Moon Y Xenobiotic-Induced Ribosomal Stress Compromises Dysbiotic Gut Barrier Aging: A One Health Perspective. Redox Biol. 2023, 59, 102565. 10.1016/J.REDOX.2022.102565. 36470131 PMC9720106

[7] Wiśniewski JR; Hein MY; Cox J; Mann MA “Proteomic Ruler” for Protein Copy Number and Concentration Estimation without Spike-in Standards. Mol. Cell Proteom. 2014, 13, 3497–3506. 10.1074/MCP.M113.037309. PMC4256500 25225357

[8] Wyant GA; Abu-Remaileh M; Frenkel EM; Laqtom NN; Dharamdasani V; Lewis CA; Chan SH; Heinze I; Ori A; Sabatini DM NUFIP1 Is a Ribosome Receptor for Starvation-Induced Ribophagy. Science 2018, 360, 751–758. 10.1126/SCIENCE.AAR2663. 29700228 PMC6020066

[9] Smriga M; Kameishi M; Uneyama H; Torii K Dietary L-Lysine Deficiency Increases Stress-Induced Anxiety and Fecal Excretion in Rats. J. Nutr. 2002, 132, 3744–3746. 10.1093/JN/132.12.3744. 12468617

[10] Fan M; Gao X; Li L; Ren Z; Lui LMW; McIntyre RS; Teopiz KM; Deng P; Cao B The Association Between Concentrations of Arginine, Ornithine, Citrulline and Major Depressive Disorder: A Meta-Analysis. Front. Psychiatry 2021, 12, 686973. 10.3389/FPSYT.2021.686973. 34867503 PMC8636832

[11] Ali-Sisto T; Tolmunen T; Viinamäki H; Mäntyselkä P; Valkonen-Korhonen M; Koivumaa-Honkanen H; Honkalampi K; Ruusunen A; Nandania J; Velagapudi V; Global Arginine Bioavailability Ratio Is Decreased in Patients with Major Depressive Disorder. J. Affect. Disord. 2018, 229, 145–151. 10.1016/J.JAD.2017.12.030. 29310063

[12] Zhang X; Eladawi MA; Ryan WG; Fan X; Prevoznik S; Devale T; Ramnani B; Malathi K; Sibille E; Mccullumsmith R; Ribosomal Dysregulation: A Conserved Pathophysiological Mechanism in Human Depression and Mouse Chronic Stress. PNAS Nexus 2023, 2, pgad299. 10.1093/PNASNEXUS/PGAD299. 37822767 PMC10563789

[13] Koopmans F; van Nierop P; Andres-Alonso M; Byrnes A; Cijsouw T; Coba MP; Cornelisse LN; Farrell RJ; Goldschmidt HL; Howrigan DP; SynGO: An Evidence-Based, Expert-Curated Knowledge Base for the Synapse. Neuron 2019, 103, 217–234.e4. 10.1016/J.NEURON.2019.05.002. 31171447 PMC6764089

[14] Flati T; Gioiosa S; Chillemi G; Mele A; Oliverio A; Mannironi C; Rinaldi A; Castrignanò T A Gene Expression Atlas for Different Kinds of Stress in the Mouse Brain. Sci. Data 2020, 7, 1–18. 10.1038/s41597-020-00772-z. 33328476 PMC7744580

[15] Kao CY; He Z; Zannas AS; Hahn O; Kühne C; Reichel JM; Binder EB; Wotjak CT; Khaitovich P; Turck CW Fluoxetine Treatment Prevents the Inflammatory Response in a Mouse Model of Posttraumatic Stress Disorder. J. Psychiatr. Res. 2016, 76, 74–83. 10.1016/J.JPSYCHIRES.2016.02.003. 26897419

[16] Peña CJ; Smith M; Ramakrishnan A; Cates HM; Bagot RC; Kronman HG; Patel B; Chang AB; Purushothaman I; Dudley J; Early Life Stress Alters Transcriptomic Patterning across Reward Circuitry in Male and Female Mice. Nat. Commun. 2019, 10, 5098. 10.1038/s41467-019-13085-6. 31704941 PMC6841985

[17] Floriou-Servou A; von Ziegler L; Stalder L; Sturman O; Privitera M; Rassi A; Cremonesi A; Thöny B; Bohacek J Distinct Proteomic, Transcriptomic, and Epigenetic Stress Responses in Dorsal and Ventral Hippocampus. Biol. Psychiatry 2018, 84, 531–541. 10.1016/j.biopsych.2018.02.003. 29605177

[18] Lori A; Maddox SA; Sharma S; Andero R; Ressler KJ; Smith AK Dynamic Patterns of Threat-Associated Gene Expression in the Amygdala and Blood. Front. Psychiatry 2019, 10, 425511. 10.3389/FPSYT.2018.00778/BIBTEX. PMC6344436 30705647

[19] Bagot RCC; Cates HMM; Purushothaman I; Lorsch ZSS; Walker DMM; Wang J; Huang X; Schlüter OMM; Maze I; Peña CJJ; Circuit-Wide Transcriptional Profiling Reveals Brain Region-Specific Gene Networks Regulating Depression Susceptibility. Neuron 2016, 90, 969–983. 10.1016/j.neuron.2016.04.015. 27181059 PMC4896746

[20] Cheng Y; Sun M; Chen L; Li Y; Lin L; Yao B; Li Z; Wang Z; Chen J; Miao Z; Ten-Eleven Translocation Proteins Modulate the Response to Environmental Stress in Mice. Cell Rep. 2018, 25, 3194–3203.e4. 10.1016/j.celrep.2018.11.061. 30540950 PMC6350936

[21] Bagot RC; Cates HM; Purushothaman I; Vialou V; Heller EA; Yieh L; LaBonté B; Peña CJ; Shen L; Wittenberg GM; Ketamine and Imipramine Reverse Transcriptional Signatures of Susceptibility and Induce Resilience-Specific Gene Expression Profiles. Biol. Psychiatry 2017, 81, 285–295. 10.1016/j.biopsych.2016.06.012. 27569543 PMC5164982

[22] Bondar N; Bryzgalov L; Ershov N; Gusev F; Reshetnikov V; Avgustinovich D; Tenditnik M; Rogaev E; Merkulova T Molecular Adaptations to Social Defeat Stress and Induced Depression in Mice. Mol. Neurobiol. 2018, 55, 3394–3407. 10.1007/S12035-017-0586-3/METRICS. 28500512

[23] Labonté B; Engmann O; Purushothaman I; Menard C; Wang J; Tan C; Scarpa JR; Moy G; Loh YHE; Cahill M; Sex-Specific Transcriptional Signatures in Human Depression. Nat. Med. 2017, 23, 1102–1111. 10.1038/nm.4386. 28825715 PMC5734943

[24] Laine MA; Trontti K; Misiewicz Z; Sokolowska E; Kulesskaya N; Heikkinen A; Saarnio S; Balcells I; Ameslon P; Greco D; Genetic Control of Myelin Plasticity after Chronic Psychosocial Stress. eNeuro 2018, 5(4). 10.1523/ENEURO.0166-18.2018. PMC6071195 30073192

[25] Misiewicz Z; Iurato S; Kulesskaya N; Salminen L; Rodrigues L; Maccarrone G; Martins J; Czamara D; Laine MA; Sokolowska E; Multi-Omics Analysis Identifies Mitochondrial Pathways Associated with Anxiety-Related Behavior. PLoS Genet. 2019, 15, e1008358. 10.1371/JOURNAL.PGEN.1008358. 31557158 PMC6762065

[26] Nollet M; Hicks H; McCarthy AP; Wu H; Möller-Levet CS; Laing EE; Malki K; Lawless N; Wafford KA; Dijk DJ; REM Sleep’s Unique Associations with Corticosterone Regulation, Apoptotic Pathways, and Behavior in Chronic Stress in Mice. Proc. Natl. Acad. Sci. U S A 2019, 116, 2733–2742. 10.1073/PNAS.1816456116/SUPPL_FILE/PNAS.1816456116.SD09.XLSX. 30683720 PMC6377491

[27] Cajigas IJ; Tushev G; Will TJ; Tom Dieck S; Fuerst N; Schuman EM The Local Transcriptome in the Synaptic Neuropil Revealed by Deep Sequencing and High-Resolution Imaging. Neuron 2012, 74, 453–466. 10.1016/J.NEURON.2012.02.036. 22578497 PMC3627340

[28] Gumy LF; Yeo GSH; Tung YCL; Zivraj KH; Willis D; Coppola G; Lam BYH; Twiss JL; Holt CE; Fawcett JW Transcriptome Analysis of Embryonic and Adult Sensory Axons Reveals Changes in MRNA Repertoire Localization. RNA 2011, 17, 85. 10.1261/RNA.2386111. 21098654 PMC3004069

[29] Poon MM; Choi SH; Jamieson CAM; Geschwind DH; Martin KC Identification of Process-Localized MRNAs from Cultured Rodent Hippocampal Neurons. J. Neurosci. 2006, 26, 13390–13399. 10.1523/JNEUROSCI.3432-06.2006. 17182790 PMC6675000

[30] Zhong J; Zhang T; Bloch LM Dendritic MRNAs Encode Diversified Functionalities in Hippocampal Pyramidal Neurons. BMC Neurosci. 2006, 7, 17. 10.1186/1471-2202-7-17/FIGURES/4. 16503994 PMC1386695

[31] Perez JD; Tom Dieck S; Alvarez-Castelao B; Tushev G; Chan ICW; Schuman EM Subcellular Sequencing of Single Neurons Reveals the Dendritic Transcriptome of GABAergic Interneurons. Elife 2021, 10, e63092. 10.7554/ELIFE.63092. 33404500 PMC7819707

[32] Zencir S; Dilg D; Rueda MP; Shore D; Albert B Mechanisms Coordinating Ribosomal Protein Gene Transcription in Response to Stress. Nucleic Acids Res. 2020, 48, 11408. 10.1093/NAR/GKAA852. 33084907 PMC7672434

[33] Shi Z; Fujii K; Kovary KM; Genuth NR; Röst HL; Teruel MN; Barna M Heterogeneous Ribosomes Preferentially Translate Distinct Subpools of MRNAs Genome-Wide. Mol. Cell 2017, 67, 71–83.e7. 10.1016/J.MOLCEL.2017.05.021. 28625553 PMC5548184

[34] Genuth NR; Barna M The Discovery of Ribosome Heterogeneity and Its Implications for Gene Regulation and Organismal Life. Mol. Cell 2018, 71, 364–374. 10.1016/j.molcel.2018.07.018. 30075139 PMC6092941

[35] Sun C; Nold A; Fusco CM; Rangaraju V; Tchumatchenko T; Heilemann M; Schuman EM The Prevalence and Specificity of Local Protein Synthesis during Neuronal Synaptic Plasticity. Sci. Adv. 2021, 7, 790–807. 10.1126/SCIADV.ABJ0790/SUPPL_FILE/SCIADV.ABJ0790_SM.PDF. PMC8448450 34533986

[36] Turrigiano G Homeostatic Synaptic Plasticity: Local and Global Mechanisms for Stabilizing Neuronal Function. Cold Spring Harb. Perspect. Biol. 2012, 4, a005736. 10.1101/CSHPERSPECT.A005736. 22086977 PMC3249629

[37] Pamplona F; Henes K; Micale V; Mauch C; Takahashi; Wotjak C Prolonged fear incubation leads to generalized avoidance behavior in mice. J. Psychiatr. Res. 2011, 45, 354–360, 10.1016/j.jpsychires.2010.06.015. 20655545

[38] Peña CJ; Kronman HG; Walker DM; Cates HM; Bagot RC; Purushothaman I; Issler O; Loh Y-HE; Leong T; Kiraly DD; Early life stress confers lifelong stress susceptibility in mice via ventral tegmental area OTX2. Science 2017, 356, 1185–1188, 10.1126/science.aan4491. 28619944 PMC5539403

[39] Mifsud KR; Reul JM Acute Stress Enhances Heterodimerization and Binding of Corticosteroid Receptors at Glucocor-ticoid Target Genes in the Hippocampus. Proc Natl Acad Sci U S A 2016, 113, 11336–11341, 10.1073/PNAS.1605246113/SUPPL_FILE/PNAS.1605246113.SD01.XLSX. 27655894 PMC5056104

[40] Berton O; McClung CA; Dileone RJ; Krishnan V; Renthal W; Russo SJ; Graham D; Tsankova NM; Bolanos CA; Rios M; Essential Role of BDNF in the Mesolimbic Dopamine Pathway in Social Defeat Stress. Science 2006, 311, 864–868, 10.1126/science.1120972. 16469931

[41] Krishnan V; Han M-H; Graham DL; Berton O; Renthal W; Russo SJ; LaPlant Q; Graham A; Lutter M; Lagace DC; Molecular Adaptations Underlying Susceptibility and Resistance to Social Defeat in Brain Reward Regions. Cell 2007, 131, 391–404, doi:10.1016/j.cell.2007.09.018. 17956738

[42] Borrow AP; Heck AL; Miller AM; Sheng JA; Stover SA; Daniels RM; Bales NJ; Fleury TK; Handa RJ Chronic variable stress alters hypothalamic-pituitary-adrenal axis function in the female mouse. Physiol. Behav. 2019, 209, 112613–112613, 10.1016/j.physbeh.2019.112613. 31299374 PMC6693655

[43] Willner P The chronic mild stress (CMS) model of depression: History, evaluation and usage. Neurobiol. Stress 2017, 6, 78–93, 10.1016/j.ynstr.2016.08.002. 28229111 PMC5314424

