# Supplementary material for: Dynamic Dysregulation of Ribosomal Protein Genes in Mouse Brain Stress Models
Source: Stresses. Author manuscript; Available in PMC 2025 Aug 15. (PMC12352335; doi:10.3390/stresses4040061)
Supplement: Supplementary Information [file NIHMS2077607-supplement-Supplementary_Information.pdf]

Supplementary information for

Dynamic Dysregulation of Ribosomal Protein Genes in Mouse Brain Stress Models

Vandana Sharma[1,2,†], and Rammohan Shukla[1,2,†]

Items		Details/Notes
**Figure S1**	:	**Differential RPG expression across various stress paradigms is organized by distinct brain regions.** The illustration indicates upregulation (red; $p < 0.05$) and downregulation (green; $p < 0.05$) of mitochondrial and cytoplasmic RPGs, with white representing RPGs that are not dysregulated. The labels on the right correspond to specific brain regions, and details for each contrast (rows in the figure) are provided in Table S4. Notably, the contrasts lose their clustering by bioproject (i.e., stress paradigms), as observed in Figure 1, and clustering by brain region does not reveal any discernible patterns, limiting the ability to draw definitive conclusions.
**Figure S2**	:	**Differential RPG expression across various stress paradigms is clustered by sex.** The details are consistent with those in Figure S1, except that the rows are now clustered based on sex, as indicated by the labels on the right. Details for each contrast (rows in the figure) are provided in Table S5. Similar to clustering by brain region, the contrasts lose their clustering by bioproject (i.e., stress paradigms). Notably, the underrepresentation of females limits the ability to draw robust conclusions.
**Table S1**	:	Summary of stress paradigms showing RPG dysregulations, along with their biological basis and relevance to depression and stress-related disorders (see below).
**Table S2**	:	Summary of stress paradigms used in mouse models, detailing the bioproject identifiers, type and frequency of stress, time elapsed from stress, targeted brain regions, mouse strain, and sex in various mouse strains. **(see Supplementary Tables; Excel sheet 1)**
**Table S3**	:	Figure 1 details are presented in a table format, listing each bioproject (stress paradigm) and its associated contrasts on the left. Colored cells display enrichment scores, with red indicating upregulation and green indicating downregulation **(see Supplementary Tables, Excel Sheet 2).**
**Table S4**	:	Figure S1 details are presented in a table format, with each contrast listed on the left. Contrasts are clustered based on brain regions (indicated by the labels on the right); however, no specific patterns emerge from this clustering. Additional details align with those provided in Table S3 **(see Supplementary Tables, Excel Sheet 3).**
**Table S5**	:	Figure S2 details are presented in table format, with each bioproject study and its associated contrasts listed on the left. Contrasts are clustered based on sex (indicated by the labels on the right); however, no specific patterns are observed in this clustering. Additional details correspond to those provided in Table S3 **(see Supplementary Tables, Excel Sheet 4).**

BNST: Bed nucleus of the stria terminalis; **NAcc:** Nucleus accumbens; **PFC:** Prefrontal Cortex; **VTA:** Ventral Tegmental Area

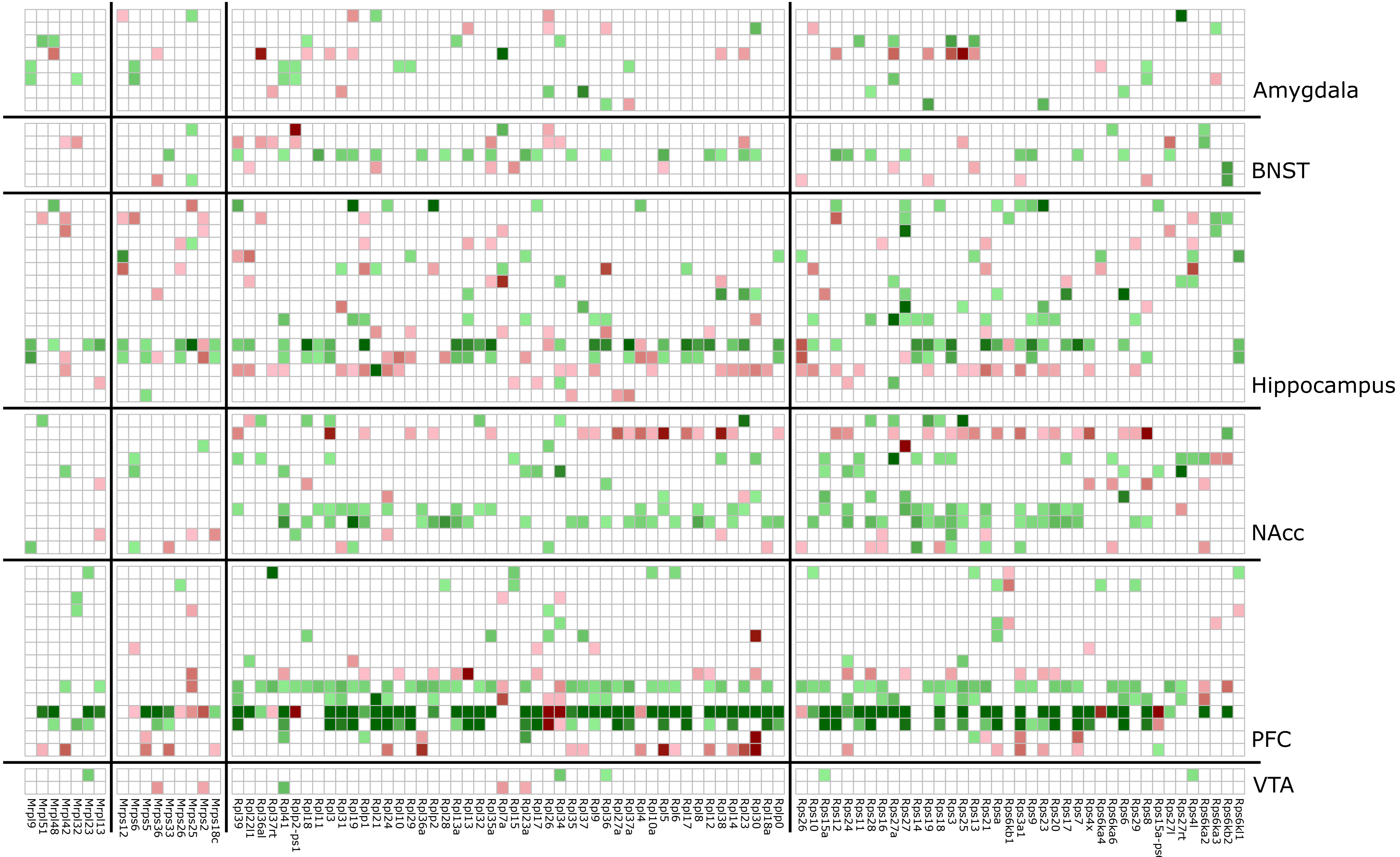
Amygdala
BNST
Hippocampus
NAcc
PFC
VTA
Mrpl9 Mrpl51 Mrpl48 Mrpl42 Mrpl32 Mrpl23 Mrpl13
Mrps12 Mrps6 Mrps5 Mrps36 Mrps33 Mrps26 Mrps25 Mrps2 Mrps18c
Rpl39 Rpl22l1 Rpl36al Rpl37rt Rpl41 Rplp2-ps1 Rpl18 Rpl11 Rpl3 Rpl31 Rpl19 Rplp1 Rpl21 Rpl24 Rpl10 Rpl29 Rpl36a Rplp2 Rpl28 Rpl13a Rpl13 Rpl32 Rpl35a Rpl7a Rpl15 Rpl23a Rpl17 Rpl26 Rpl34 Rpl35 Rpl37 Rpl9 Rpl36 Rpl27a Rpl37a Rpl4 Rpl10a Rpl5 Rpl6 Rpl17 Rpl8 Rpl12 Rpl38 Rpl14 Rpl23 Rpl30 Rpl18a Rplp0
Rps26 Rps10 Rps15a Rps12 Rps24 Rps11 Rps28 Rps16 Rps27a Rps27 Rps14 Rps19 Rps18 Rps3 Rps25 Rps13 Rps21 Rpsa Rps6kb1 Rps3a1 Rps9 Rps23 Rps20 Rps17 Rps7 Rps4x Rps6ka4 Rps6ka6 Rps6 Rps29 Rps8 Rps15a-ps6 Rps27l Rps27rt Rps4l Rps6ka2 Rps6ka3 Rps6kb2 Rps6kl1

Figure S1

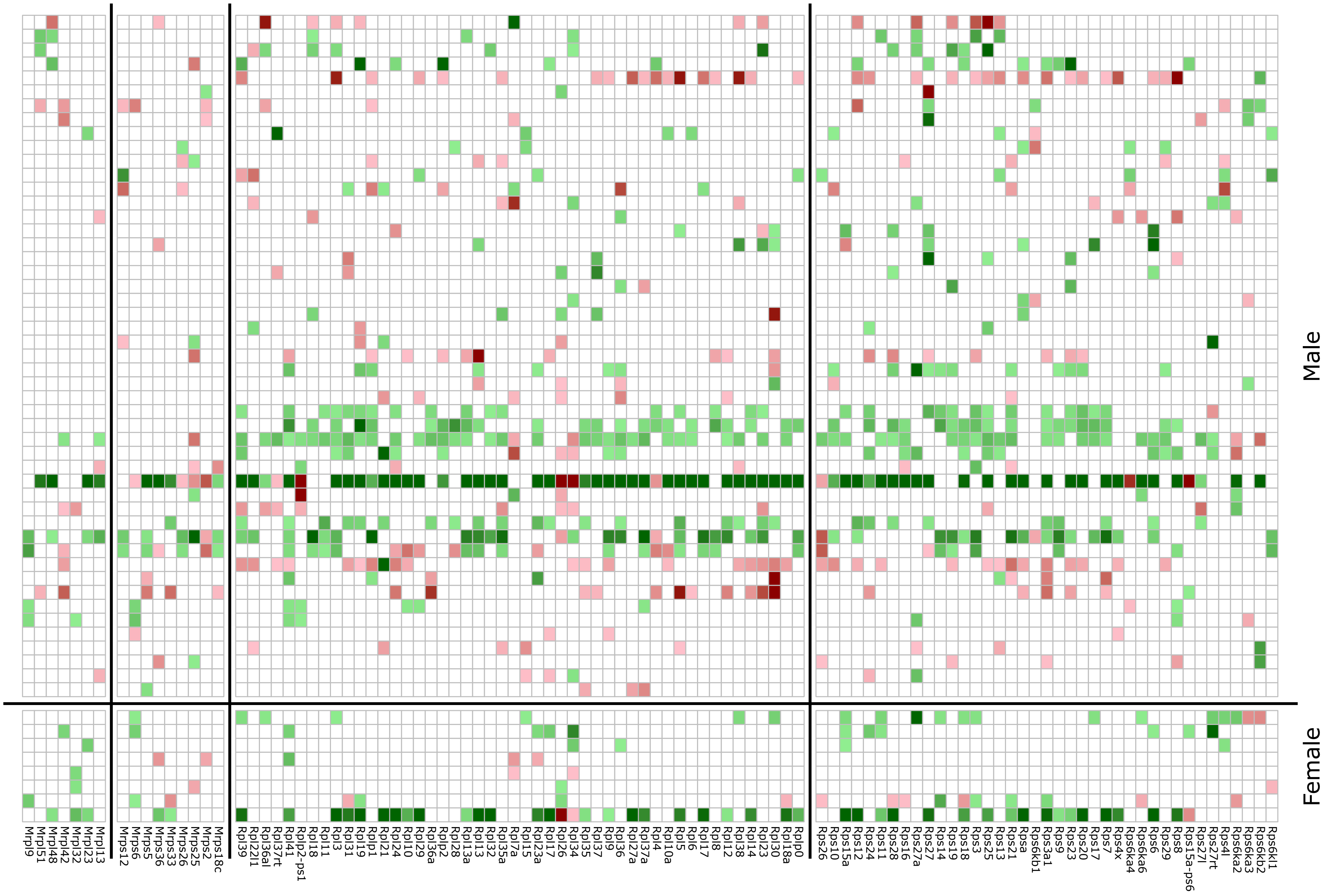
Male
Female
Mrpl9 Mrpl51 Mrpl48 Mrpl42 Mrpl32 Mrpl23 Mrpl13
Mrps12 Mrps6 Mrps5 Mrps36 Mrps33 Mrps26 Mrps25 Mrps2 Mrps18c
Rpl39 Rpl22l1 Rpl36al Rpl37rt Rpl41 Rplp2-ps1 Rpl18 Rpl11 Rpl3 Rpl31 Rpl19 Rplp1 Rpl21 Rpl24 Rpl10 Rpl29 Rpl36a Rplp2 Rpl28 Rpl13a Rpl13 Rpl32 Rpl35a Rpl7a Rpl15 Rpl23a Rpl17 Rpl26 Rpl34 Rpl35 Rpl37 Rpl9 Rpl36 Rpl27a Rpl37a Rpl4 Rpl10a Rpl5 Rpl6 Rpl7 Rpl8 Rpl12 Rpl38 Rpl14 Rpl23 Rpl30 Rpl18a Rplp0
Rps26 Rps10 Rps15a Rps12 Rps24 Rps11 Rps28 Rps16 Rps27a Rps27 Rps14 Rps19 Rps18 Rps3 Rps25 Rps13 Rps21 Rpsa Rps6kb1 Rps3a1 Rps9 Rps23 Rps20 Rps17 Rps7 Rps4x Rps6ka4 Rps6ka6 Rps6 Rps29 Rps8 Rps15a-ps6 Rps27rt Rps27l Rps4l Rps6ka2 Rps6ka3 Rps6kb2 Rps6kl1

Figure S2

Stress Paradigm	Type	Biological Basis [Reference]
Electric foot shock (2 sec, 1.5 mA)	Acute	This model is characterized by Long-term maintenance of generalized fear, avoidance and hyperarousal as well as altered hippocampus volume [1,2].
Early life stress; Subthreshold variable stress	Chronic	Early life stress increases risk for depression by heightening sensitivity to stressful events later in life, with early stress exerting prominent effects on the brain's reward circuitry across species [3,4].
Restraint (30 min), Cold swim (6 min)	Acute	Activates different neuronal networks; shows rapid binding of both glucocorticoid and mineralocorticoid receptors to glucocorticoid response elements [5,6].
Auditory fear conditioning; Immobilization plus Auditory fear conditioning	Acute	An amygdala-dependent rodent model of threat-exposure, providing insight into the physiological processes underlying response to trauma [7].
Social defeat (10 d)	Chronic	A model that significantly alters the motivation for social interactions in rodents, effectively mimicking the symptomatology of stress-related disorders such as PTSD and depression [8–10].
Restraint (15 d, 2 hr/d)	Chronic	This model is particularly relevant for studying the long-term biological mechanisms of stress-induced depression, including changes in hippocampal plasticity, neuroinflammation, and monoaminergic signaling [11].
Social defeat (10 d) followed by stress re-exposure after 28 days	Chronic	The CSDS phenotype merges by 48h. 28 days post CSDS is used to study the persistence of susceptible and resilient phenotypes [9,12].
Social defeat (10 or 30 d)	Chronic	Glucocorticoid-sensitive genes are stimulated on the 10th day of stress, but these genes stop responding to the elevated corticosterone level by the 30th day of stress [13].
Variable stress (mild foot shock, tail suspension and restraint, alternated over 21 d, 1 hr/d)	Chronic	This model simulates the effects of long-term stress in humans, leading to behavioral changes that resemble symptoms of anxiety and depression. It also replicates alterations in the HPA axis commonly observed in human depression [14,15].
Unpredictable mild stress (various mild stressors, alternated daily over 9 weeks)	Chronic	Elicits a broad range of physiological and ethological changes that are consistent with symptoms of major depressive disorder and predicts the efficacy of antidepressant treatments [16,17].

Table S1: Summary of stress paradigms with biological basis and relevance to depression and stress-related disorders. The table categorizes various acute and chronic stress paradigms analyzed this study (Figure 1), highlighting their biological basis. These paradigms model different aspects of stress-related psychopathology, including PTSD, depression, and anxiety, and provide insights into physiological and molecular mechanisms underlying stress responses.

Reference:

1. Pamplona, F.A.; Henes, K.; Micale, V.; Mauch, C.P.; Takahashi, R.N.; Wotjak, C.T. Prolonged Fear Incubation Leads to Generalized Avoidance Behavior in Mice. *J Psychiatr Res* 2011, *45*, 354–360, doi:10.1016/J.JPSYCHIRES.2010.06.015.
2. Kao, C.Y.; He, Z.; Zannas, A.S.; Hahn, O.; Kühne, C.; Reichel, J.M.; Binder, E.B.; Wotjak, C.T.; Khaitovich, P.; Turck, C.W. Fluoxetine Treatment Prevents the Inflammatory Response in a Mouse Model of Posttraumatic Stress Disorder. *J Psychiatr Res* 2016, *76*, 74–83, doi:10.1016/J.JPSYCHIRES.2016.02.003.
3. Peña, C.J.; Smith, M.; Ramakrishnan, A.; Cates, H.M.; Bagot, R.C.; Kronman, H.G.; Patel, B.; Chang, A.B.; Purushothaman, I.; Dudley, J.; et al. Early Life Stress Alters Transcriptomic Patterning across Reward Circuitry in Male and Female Mice. *Nature Communications 2019 10:1* 2019, *10*, 1–13, doi:10.1038/s41467-019-13085-6.
4. Peña, C.J.; Kronman, H.G.; Walker, D.M.; Cates, H.M.; Bagot, R.C.; Purushothaman, I.; Issler, O.; Eddie Loh, Y.H.; Leong, T.; Kiraly, D.D.; et al. Early Life Stress Confers Lifelong Stress Susceptibility in Mice via Ventral Tegmental Area OTX2. *Science* 2017, *356*, 1185, doi:10.1126/SCIENCE.AAN4491.
5. Floriou-Servou, A.; von Ziegler, L.; Stalder, L.; Sturman, O.; Privitera, M.; Rassi, A.; Cremonesi, A.; Thöny, B.; Bohacek, J. Distinct Proteomic, Transcriptomic, and Epigenetic Stress Responses in Dorsal and Ventral Hippocampus. *Biol Psychiatry* 2018, *84*, 531–541, doi:10.1016/j.biopsych.2018.02.003.
6. Mifsud, K.R.; Reul, J.M.H.M. Acute Stress Enhances Heterodimerization and Binding of Corticosteroid Receptors at Glucocorticoid Target Genes in the Hippocampus. *Proc Natl Acad Sci U S A* 2016, *113*, 11336–11341, doi:10.1073/PNAS.1605246113/SUPPL_FILE/PNAS.1605246113.SD01.XLSX.
7. Lori, A.; Maddox, S.A.; Sharma, S.; Andero, R.; Ressler, K.J.; Smith, A.K. Dynamic Patterns of Threat-Associated Gene Expression in the Amygdala and Blood. *Front Psychiatry* 2019, *10*, 425511, doi:10.3389/FPSYT.2018.00778/BIBTEX.
8. Berton, O.; McClung, C.A.; DiLeone, R.J.; Krishnan, V.; Renthal, W.; Russo, S.J.; Graham, D.; Tsankova, N.M.; Bolanos, C.A.; Rios, M.; et al. Essential Role of BDNF in the Mesolimbic Dopamine Pathway in Social Defeat Stress. *Science (1979)* 2006, *311*, 864–868, doi:10.1126/SCIENCE.1120972/SUPPL_FILE/BERTON.SOM.PDF.
9. Krishnan, V.; Han, M.H.; Graham, D.L.; Berton, O.; Renthal, W.; Russo, S.J.; LaPlant, Q.; Graham, A.; Lutter, M.; Lagace, D.C.; et al. Molecular Adaptations Underlying Susceptibility and Resistance to Social Defeat in Brain Reward Regions. *Cell* 2007, *131*, 391–404, doi:10.1016/J.CELL.2007.09.018.
10. Bagot, R.C.; Cates, H.M.; Purushothaman, I.; Vialou, V.; Heller, E.A.; Yieh, L.; LaBonté, B.; Peña, C.J.; Shen, L.; Wittenberg, G.M.; et al. Ketamine and Imipramine Reverse Transcriptional Signatures of Susceptibility and Induce Resilience-Specific Gene Expression Profiles. *Biol Psychiatry* 2017, *81*, 285–295, doi:10.1016/j.biopsych.2016.06.012.
11. Cheng, Y.; Sun, M.; Chen, L.; Li, Y.; Lin, L.; Yao, B.; Li, Z.; Wang, Z.; Chen, J.; Miao, Z.; et al. Ten-Eleven Translocation Proteins Modulate the Response to Environmental Stress in Mice. *Cell Rep* 2018, *25*, 3194-3203.e4, doi:10.1016/j.celrep.2018.11.061.
12. Bagot, R.C.C.; Cates, H.M.M.; Purushothaman, I.; Lorsch, Z.S.S.; Walker, D.M.M.; Wang, J.; Huang, X.; Schlüter, O.M.M.; Maze, I.; Peña, C.J.J.; et al. Circuit-Wide Transcriptional Profiling Reveals Brain Region-Specific Gene Networks Regulating Depression Susceptibility. *Neuron* 2016, *90*, 969–983, doi:10.1016/j.neuron.2016.04.015.
13. Bondar, N.; Bryzgalov, L.; Ershov, N.; Gusev, F.; Reshetnikov, V.; Avgustinovich, D.; Tenditnik, M.; Rogaev, E.; Merkulova, T. Molecular Adaptations to Social Defeat Stress and Induced Depression in Mice. *Mol Neurobiol* 2018, *55*, 3394–3407, doi:10.1007/S12035-017-0586-3/METRICS.
14. Borrow, A.P.; Heck, A.L.; Miller, A.M.; Sheng, J.A.; Stover, S.A.; Daniels, R.M.; Bales, N.J.; Fleury, T.K.; Handa, R.J. Chronic Variable Stress Alters Hypothalamic-Pituitary-Adrenal Axis Function in the Female Mouse. *Physiol Behav* 2019, *209*, 112613, doi:10.1016/J.PHYSBEH.2019.112613.

15. Labonté, B.; Engmann, O.; Purushothaman, I.; Menard, C.; Wang, J.; Tan, C.; Scarpa, J.R.; Moy, G.; Loh, Y.H.E.; Cahill, M.; et al. Sex-Specific Transcriptional Signatures in Human Depression. *Nat Med* 2017, *23*, 1102, doi:10.1038/NM.4386.
16. Nollet, M.; Hicks, H.; McCarthy, A.P.; Wu, H.; Möller-Levet, C.S.; Laing, E.E.; Malki, K.; Lawless, N.; Wafford, K.A.; Dijk, D.J.; et al. REM Sleep's Unique Associations with Corticosterone Regulation, Apoptotic Pathways, and Behavior in Chronic Stress in Mice. *Proc Natl Acad Sci U S A* 2019, *116*, 2733–2742, doi:10.1073/PNAS.1816456116/SUPPL_FILE/PNAS.1816456116.SD09.XLSX.
17. Willner, P. The Chronic Mild Stress (CMS) Model of Depression: History, Evaluation and Usage. *Neurobiol Stress* 2016, *6*, 78–93, doi:10.1016/J.YNSTR.2016.08.002.
